# Contact Tracing Activities during the Ebola Virus Disease Epidemic in Kindia and Faranah, Guinea, 2014

**DOI:** 10.3201//eid2111.150684

**Published:** 2015-11

**Authors:** Meredith G. Dixon, Melanie M. Taylor, Jacob Dee, Avi Hakim, Paul Cantey, Travis Lim, Hawa Bah, Sékou Mohamed Camara, Clement B. Ndongmo, Mory Togba, Leonie Yvonne Touré, Pepe Bilivogui, Mohammed Sylla, Michael Kinzer, Fátima Coronado, Jon Eric Tongren, Mahesh Swaminathan, Lise Mandigny, Boubacar Diallo, Thomas Seyler, Marc Rondy, Guénaël Rodier, William A. Perea, Benjamin Dahl

**Affiliations:** US Centers for Disease Control and Prevention, Atlanta, Georgia, USA (M.G. Dixon, M.M. Taylor, J. Dee, A. Hakim, P. Cantey, T. Lim, M. Kinzer, F. Coronado, M. Swaminathan, B. Dahl);; World Health Organization, Brazzaville, Congo (H. Bah, S.M. Camara);; US Centers for Disease Control and Prevention, Lusaka, Zambia (C.B. Ndongmo);; Ministry of Health, Guinea (M. Togba, L.Y. Touré, P. Bilivogui, M. Sylla);; US Centers for Disease Control and Prevention, Kigali, Rwanda (J.E. Tongren);; World Health Organization, Geneva, Switzerland (L. Mandigny, B. Diallo, G. Rodier, W.A. Perea);; EpiConcept, Paris, France; World Health Organization Ebola Response Team, Conakry, Guinea (T. Seyler, M. Rondy)

**Keywords:** Ebola, viruses, contact tracing, epidemic control, Guinea, Ebola virus disease

## Abstract

The largest recorded Ebola virus disease epidemic began in March 2014; as of July 2015, it continued in 3 principally affected countries: Guinea, Liberia, and Sierra Leone. Control efforts include contact tracing to expedite identification of the virus in suspect case-patients. We examined contact tracing activities during September 20–December 31, 2014, in 2 prefectures of Guinea using national and local data about case-patients and their contacts. Results show less than one third of case-patients (28.3% and 31.1%) were registered as contacts before case identification; approximately two thirds (61.1% and 67.7%) had no registered contacts. Time to isolation of suspected case-patients was not immediate (median 5 and 3 days for Kindia and Faranah, respectively), and secondary attack rates varied by relationships of persons who had contact with the source case-patient and the type of case-patient to which a contact was exposed. More complete contact tracing efforts are needed to augment control of this epidemic.

## Abstract 2

Thorough case identification and contact tracing are necessary to end this epidemic.

During March 23, 2014–July 8, 2015, Guinea reported 3,748 Ebola virus disease (EVD) cases and 2,499 EVD-related deaths (*1*), as part of what is the largest reported EVD epidemic to date (*2*). Thorough case identification and contact tracing are necessary to end this epidemic (*3*). Contact tracing involves locating all persons who have been exposed to someone infected with Ebola virus (case-patients) or their body fluids and monitoring them daily for EVD symptoms during the 3 weeks after the last exposure (*4*). This tracing permits immediate identification and isolation of symptomatic contacts (suspected case-patients). Incomplete contact tracing and delayed time to isolation of suspected case-patients may result in transmission of EVD to others in the community, perpetuating the epidemic.

Excluding Conakry, the capital, Guinea is divided into 33 prefectures, which are subdivided into >300 subprefectures; these divisions are large and smaller administrative governmental units, respectively. Of all Ebola cases nationwide, 3.0% and 1.9% have been identified in Kindia and Faranah (Organisation Mondiale de la Sante, unpub. data), respectively_,_ where respective populations are 4.1% and 2.6% of the national population (Institut National de la Statistique, Guinée, unpub. data). At the time of data collection, neither prefecture had its own Ebola treatment unit (ETU) or laboratory with Ebola virus testing capabilities; suspected case-patients were transported by ambulance to the nearest ETU, which was at minimum a 3-hour drive from either prefecture (Figure 1). We conducted a retrospective review of case and contact tracing data collected from a convenience sample from 2 Guinea prefectures, Kindia and Faranah, during the EVD epidemic response from September 20 through December 31, 2014. We provide descriptive analyses of case and contact tracing for these 2 prefectures to identify gaps in reporting and the yield of contact tracing; we also propose actions for improvement of the contact tracing process.

**Figure 1:**
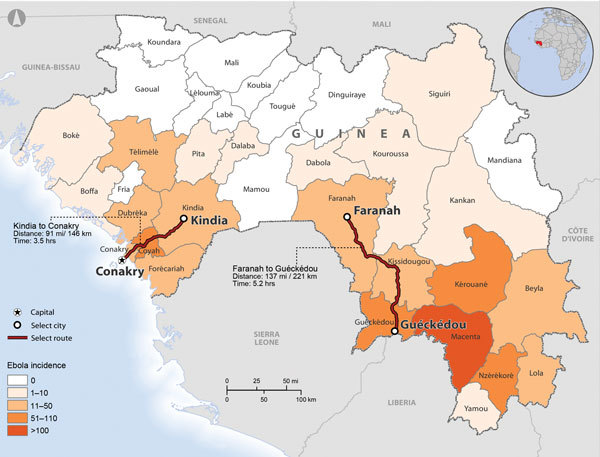
Ebola virus disease incidence (confirmed cases per 100,000 population), by prefecture, Guinea, 2014. Distances and driving times for the transport of suspect case-patients from Kindia or Faranah to the nearest Ebola treatment unit are shown (red lines). Data sources: Guinea Ministry of Health; Guinea Ministry of Planning; Database of Global Administrative Areas (GADM); Europa.

## Methods

### Case Identification

EVD cases in Guinea are categorized into 1 of 3 case definitions modified from World Health Organization recommendations: 1) suspected case (in a living person with fever and >3 of these symptoms: headache, anorexia, lethargy, aching muscles or joints, difficulty breathing, vomiting, diarrhea, stomach pain, difficulty swallowing, hiccups; or with fever and a history of contact with a person with hemorrhagic fever or a dead or sick animal; or with unexplained bleeding); 2) probable case (in a deceased person who otherwise met the suspect case definition and has an epidemiologic link to a confirmed or probable case); or 3) confirmed case (suspected or probable case that also has laboratory confirmation) (*5*). Cases are reported by using a standardized case reporting form, data from which are submitted to the national viral hemorrhagic fever (VHF) case database.

Cases from the 2 prefectures were identified and cross-referenced between the national VHF case database and the prefecture case database. Demographic information (sex, age) and case classification (confirmed, probable) were abstracted. Because this investigation was part of a public health response and considered to be nonresearch, it was not subject to US Centers for Disease Control and Prevention Institutional Review Board review.

### Contact Identification

To identify and register contacts of persons infected with EVD, prefecture public health officials and ETU staff interviewed case-patients, their families, and community members and documented resulting information on standardized contact registration forms (*6*). A contact is defined as someone at risk for infection with EVD because he or she has slept in the same household as a confirmed or probable EVD case-patient, had direct physical contact with the case-patient during that person’s illness, had direct physical contact with the body of a case-patient at a funeral or during burial preparation, touched the body fluids of a case-patient during illness, touched the case-patient’s clothes or linens, or is an infant breastfed by the case-patient (*6*). Demographic data of contacts (name, age, sex, relationship to the presumed source case-patient, and prefecture and subprefecture of residence) and daily follow-up data (presence or absence of symptoms) were obtained through use of standardized contact tracing forms (*6*), which populated a prefecture contact database.

We performed demographic descriptive analyses using nonduplicated contact data; the individual person was the unit of analysis. We performed other nondemographic descriptive analyses using contact event data; the contact event was the unit of analysis because a single contact may have had contact with several case-patients, resulting in several contact events per person. The case and contact databases may show differing numbers of source cases because contacts in a prefecture might have contacted source case-patients in another prefecture and data quality issues could exist. The date of isolation was the date a suspected case-patient was transported to an ETU. We created the following definitions: time to isolation (days) was calculated by subtracting the date of first symptom onset from the date of isolation; family/household member was anyone related by blood or marriage or who lived in the same household as the case-patient or was described as being a caregiver, excluding health care workers; safe burial was a burial with placement of the body in an impermeable bag and interment by a team wearing personal protective equipment (*7*)_._ Secondary attack rate was calculated as the proportion of new cases among contact events × 100 (*8*). Because variables had nonparametric distributions, medians were analyzed. Relative risks were used to quantify the risk for becoming a secondary case-patient after exposure to a case-patient by relationship status or a case-patient by epidemiologic case classification. We used χ^2^ tests to measure associations between categorical variables; specifically, to compare attack rates among family members and non–family members and among contacts to confirmed versus probable cases. A p value of ≤0.05 was considered statistically significant. Epidemiologic weeks were in accordance with those designated by in-country situation reports. 

## Results

### Kindia

#### Cases

**Figure 2:**
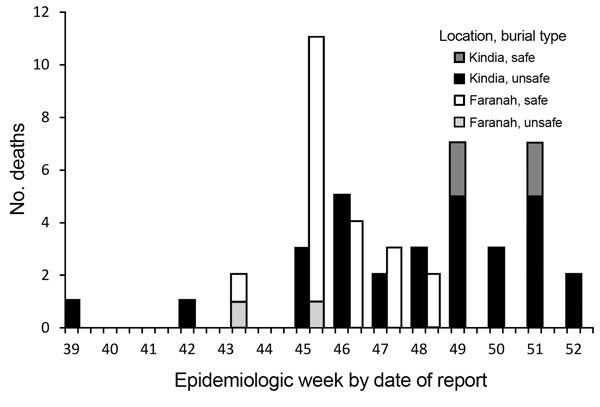
Community deaths by burial type for case-patients with confirmed and probable cases of Ebola virus disease in Kindia and Faranah, by epidemiological week, Guinea, 2014. Safe burial was defined as placement of the body in an impermeable bag and interment by a team wearing personal protective equipment (*9*).

During September 20–December 31, 2014, a total of 90 EVD cases were reported in Kindia; 63 (70%) were confirmed and 27 (30%) probable cases (Table 1). The median case-patient age was 35 (interquartile range [IQR] 20–50) years; 21 (23.3%) case-patients were <18 years of age, and 52 (57.8%) were female. No case-patients were health care workers. Case-patients resided permanently in 23 villages in 7 subprefectures of Kindia. Median time to isolation for suspect case-patients was 5 (IQR 3–7) days; this time varied by epidemiologic week. Seventy-one (78.9%) case-patients died; of those, 35 (49.1%) died in the community, of whom 30 (85.7%) underwent unsafe burial. The number of community deaths per epidemiologic week fluctuated (range 1–7) and peaked during weeks 49 and 51 (Figure 2).

**Table 1:** Demographic characteristics of Ebola virus disease case-patients in 2 prefectures, Guinea, September 20–December 31, 2014*

Characteristic	Prefecture	
	Kindia, n = 90	Faranah, n = 62
Case classification, no. (%) patients		
Confirmed	63 (70.0)	39 (62.9)
Probable	27 (30.0)	23 (37.1)
Registered as contacts before case identification, no. (%) patients	28 (31.1)	17 (27.4)
Age, y		
Median	35.0	30.0
IQR	20.0–50.0	14.0–47.0
<18 y, no. (%)	21 (23.3)	19 (30.6)
Female sex, no. (%) patients	52 (57.8)	33 (53.2)
Villages, no.	23	11
Subprefectures, no.	7	4
Median time to isolation, d (IQR)	5 (3­­–7)	3 (1–6)
Final outcome, no. (%) patients		
Deceased	71 (78.9)	52 (83.9)
Place of death, no. (%) patients		
Ebola treatment unit	36 (50.7)	28 (53.8)
Community	35 (49.1)	24 (46.1)
Burial type for community deaths, no. (%) patients†		
Safe	5 (14.3)	20 (90.9)
Unsafe	30 (85.7)	2 (9.1)
Case-patients for whom contacts are registered, no. (%)	35 (38.9)	20 (32.2)

*Data in this table originate from the prefecture case database. The variables “registered as contacts before case identification” and “No. (%)” were created in the prefecture case database by cross-referencing with the contact database. IQR, interquartile range. †Burial data from Faranah missing for 2 case-patients.

#### Contact Tracing

Twenty-eight (31.1%) of 90 case-patients in Kindia were identified as contacts and registered in the contact database before being identified as a case-patient (Table 1). Fifty-five (61.1%) of 90 case-patients had no contacts listed in the contact database (Table 1). Thirty-five (38.9%) of the 90 case-patients in the case database had contacts listed in the contact database; 25 (71.4%) were confirmed and 10 (28.6%) had probable cases. For the 35 case-patients for whom contacts were registered, the median number of contacts per case was 16 (IQR 11.2–28.0) (Table 2).

**Table 2:** Demographic characteristics of contacts of Ebola virus disease case-patients in 2 prefectures, Guinea, September 20-December 31, 2014*

Characteristic	Prefecture	
	Kindia	Faranah
No. contacts	1,137	289
No. source case-patients	50	27
No. contact events	1,233	317
Median no. contacts per case-patient (IQR)	16 (11.2–28)	9 (5.5–15.5)
Age, y		
Median	22	20
IQR	10–40	8–35
<18 y, no. (%)	450 (39.6)	124 (42.9)
Female sex, no. (%)	611 (53.7)	146 (50.5)
Village, no.	58	24
Subprefecture	10	8
Relationship to source case-patient, no. (%)		
Family/household member	470 (41.3)	152 (52.6)
Neighbor	464 (40.8)	6 (2.1)
Health care worker	22 (1.9)	0
Teacher	1 (0.1)	0
Other	17 (1.5)	39 (13.5)
No data	163 (14.3)	92 (31.8)

*Data in this table are from the contact databases; source case-patients listed here are not necessarily the same as case-patients listed in Table 1. IQR, interquartile range.

The Kindia contact database contained data on 1,137 contacts of 50 source case-patients (29 confirmed, 11 probable; 10 had unknown classification) (Table 2). Some of the 50 source case-patients did not reside in Kindia but were source case-patients of contacts followed in Kindia. The median age of contacts was 22 (IQR 10–40) years; 450 (39.6%) were <18 years of age, and 611 (53.7%) were female. Family or household members accounted for 470 (41.3%) contacts.

Among the 1,137 contacts and case-patients with 50 source case-patients, 1,233 contact-events occurred; 26 contacts became ill with EVD, for an overall secondary attack rate of 2.1%. Nineteen (73.1%) of these patients had confirmed cases and 7 (26.9%) had probable cases. Eighteen of 829 contacts exposed to a confirmed case-patient and 7 of 226 contacts exposed to a patient who had a probable case became infected, for secondary attack rates of 2.2% and 3.1%, respectively (p = 0.4). Data on the epidemiologic classification of the source case were missing for 1 secondary case-patient.

The median age of secondary case-patients was 28 (IQR 9–60) years; 7 (26.9%) were <18 years of age, and 15 (57.7%) were female. The secondary attack rate was 4.2% (20 cases among 470 contact-events) when the contact was a family or household member of the source case-patient but only 0.4% (2 of 507) when the contact was not a family or household member (relative risk 10.8, 95% CI 2.5–45.9). There was no statistically significant difference in the risk for becoming a secondary case-patient for contacts who were <18 years of age or according to sex. No contacts who were traditional healers or health care workers became secondary case-patients.

### Faranah

#### Cases

During September 25–December 12, 2014, a total of 62 EVD cases were reported in Faranah; 39 (62.9%) were confirmed and 23 (37.1%) probable cases (Table 1). The median case-patient age was 30 (IQR 14–47) years; 19 (30.1%) case-patients were <18 years of age, and 33 (53.2%) were female. One case-patient was a health care worker. Patients resided permanently in 11 villages in 4 subprefectures of Faranah. Median time to isolation for suspect case-patients was 3 (IQR 1–6) days; this time varied by epidemiologic week. Fifty-two (83.8%) case-patients died; of those, 24 (46.1%) died in the community, of whom 2 (9.1%) underwent unsafe burial (burial data were missing for 2). The number of community deaths per epidemiologic week decreased over time after week 45 (Figure 2).

#### Contact Tracing

Seventeen (27.4%) of 62 case-patients in Faranah were identified as contacts and registered in the contact database before being identified as a case-patient (Table 1). No contacts were listed in the contact database for 39 (62.9%) of 62 cases, and contact data were missing for 3. Of the 20 (32.2%) cases for which contacts were listed in the contact database, 10 (50.0%) were confirmed and 10 (50.0%) were probable cases. For the 20 case-patients for whom contacts were registered, the median number of contacts per case-patient was 9 (IQR 5.5–15.5) (Table 2).

The Faranah contact database contained data for 289 contacts of 27 source case-patients (8 confirmed, 10 probable; 9 had unknown epidemiologic classification) (Table 2). The median age of contacts was 20 (IQR 8–35) years; 124 (42.9%) were <18 years of age, and 146 (50.5%) were female. Family or household members accounted for 152 (52.6%) contacts.

Among 289 contacts and 27 source case-patients, 317 contact events occurred. Twenty-five contacts became ill with EVD; the overall secondary attack rate was 7.9%. Of these patients, 21 (84%) had confirmed cases and 4 (16%) had probable cases. Seven of 92 contacts exposed to a confirmed case-patient and 13 of 132 contacts exposed to a patient who had a probable case became infected, accounting for secondary attack rates of 7.6% and 9.8%, respectively (p = 0.8). The median age of secondary case-patients was 30 (IQR 16–45) years; 8 (32%) were <18 years of age, and 11 (44%) were female. The secondary attack rate was 12.3% (19 cases among 154 contact-events) when the contact was a family or household member of a case-patient and 4.8% (3 cases among 63 contact events) when the contact was not a family or household member of a case-patient (relative risk 2.6, 95% CI 0.6–10.8). There was also no statistically significant difference in the risk for having a confirmed case by persons <18 years of age or by sex. No transmission was reported between the health care worker who had a confirmed case and contacts, although at the time of data collection, contacts had not completed their 21-day follow-up review.

## Discussion

This evaluation of 2 EVD-affected prefectures of Guinea documents 2 major gaps in contact tracing activities: 1) most case-patients were not previously registered and followed up on as contacts before case identification, and 2) most case-patients, once identified, had zero contacts registered, so any contacts they had were not properly investigated. Time to isolation of suspect case-patients was suboptimal in both prefectures. Many deaths occurred in the community, and a high percentage of unsafe burials occurred in Kindia. Somewhat higher secondary attack rates occurred among contacts who were family or household members of their source case-patient and among contacts of probable case-patients.

One third of case-patients were previously identified and followed up on as contacts before onset and confirmation of EVD. Without identification of all contacts of a case-patient, it is not possible to provide adequate follow-up and ensure prompt isolation if those contacts become symptomatic. Suspected case-patients that are not isolated from the community, if infected, can transmit EVD and thus serve as reservoirs of infection. In addition, we identified suboptimal time for isolation of persons who were suspect case-patients. Not having been followed up on initially as registered contacts may have contributed to this finding. Although this evaluation was not sufficiently designed to measure the contribution of community or individual reluctance to participate in contact tracing or time to isolation of suspect case-patients, situation reports from Kindia identify these factors as barriers to contact tracing activities. Further, a recent report noted that violence related to response control efforts has been particularly problematic in Guinea, compared with Liberia and Sierra Leone, and has been a barrier to community access (*9*). These barriers to implementation of optimal disease control interventions need further evaluation to identify effective community engagement strategies.

Similarly, only one third of cases had contacts registered and followed up on according to contact tracing guidelines. There are multiple reasons for which a contact may not be registered or followed up on, including: case-patients are sometimes incapacitated or die before providing complete data; interviewed case-patients and community members might not disclose complete contact data; and families and communities might not permit public health officials in their homes or communities for contact tracing purposes because of fear or stigma. In addition, identified contacts may not cooperate with public health officials and community health agents may cease efforts to engage uncooperative or threatening contacts.

Probable cases and community deaths, especially among known contacts, represent missed opportunities for case confirmation and isolation in the response effort. Unsafe burials perpetuate EVD transmission because persons who have EVD are highly viremic before death. In Kindia and Faranah, we identified a high percentage (49% and 46%, respectively) of community deaths; a high percentage of probable cases (37% and 30%) as compared to a Guinea national proportion of 11.8% (*1*); and a high proportion of unsafe burials (86%) in Kindia. Evidence from previous outbreaks reveals a relative risk of 2.1 for virus transmission from a deceased EVD case-patient to adult family members when controlling for direct contact and exposure to body fluids (*10*). Unsafe burials have been associated with large local outbreaks; in December 2014, one unsafe burial in Guinea led to 85 confirmed cases (*11*). Both prefectures reported higher attack rates among contacts exposed to a probable versus a confirmed case, although these differences were small and not statistically significant. Probable cases are likely to be underreported, so the actual percentage of these among all cases is likely higher than reported here.

Higher secondary attack rates among family and household members of case-patients demonstrate the need for larger analyses to assess the effect of the relationship between a contact and source case-patient on disease transmission risk. For this large and complex response, which at times has been hampered by human resources limitations, it would be useful to know if stratification of contacts could be performed to more efficiently focus response efforts on those most at risk. Conversely, enrolling persons who are at minimal risk might increase community reticence and overburden local contact tracing teams. Our investigation found that a large proportion of registered contacts (47.6%) in Kindia were neighbors, whereas a small proportion (2.1%) of contacts in Faranah were neighbors. Although the relationship between family status and attack rate should be further studied, contact tracing teams should adhere to strict use of the contact definition to ensure follow-up of those truly at risk, while minimizing unnecessary follow-up of persons who do not fit the contact definition.

Limitations include, but are not restricted to, the factors provided herein. Data collection during an emergency response, especially of this scope and magnitude, is difficult. There were missing data, data of poor quality, and data that may have been in paper format only. For example, no contacts listed in the contact database may mean that no data was collected electronically or that the task was never performed. These issues were compounded by data management challenges, most specifically the lack of unique identifiers with which to link source case-patients and contacts. Thus, manual linkage of case and contacts was necessary; this task was time-consuming, onerous, and had the potential to introduce errors because of multiple data sources and common use of a small number of given and family names within a restricted geographic region. Also, because of inconsistent data flow and incompleteness, the national contact database was not used in this analysis. Thus, additional contacts named in other prefectures could have been missed, which may contribute to lower attack rates in this analysis. 

Another limitation is underreporting. Field investigations in early fall 2014 across 5 prefectures in Guinea determined that probable EVD cases comprised 30%–50% of cases (M.G. Dixon, unpub. data), whereas situation reports from October 2014–April 2015 showed that probable cases represented only 10%–12% of all cases nationwide (*1*). This difference in proportion of probable cases in overlapping time periods could mean that a higher percentage of cases are being tested and confirmed but more likely represents underreporting of probable cases. This underreporting could artificially lower the attack rates discussed here. Similarly, the inclusion of source case-patients for whom epidemiologic classification is missing may also underestimate the effects of secondary transmission on the basis of epidemiologic source case classification being probable. 

In addition, sample size limited our ability to show statistical significance for some associations. These results represent contact tracing activities from 2 prefectures of Guinea and should be generalized with caution.

The case and contact tracing descriptive analyses for these 2 prefectures in Guinea demonstrate that most case-patients were not previously registered and observed as contacts before case identification and that most case-patients had no contacts that were registered and followed up on. Individual control measures should be performed more completely to end the epidemic in Guinea. Every EVD case-patient not previously observed as a contact represents an unidentified chain of transmission. Every contact not identified or followed up on represents a possible transmission opportunity. Whereas no single control measure in this epidemic in Guinea will achieve the goal of “Getting to Zero” (http://www.unicef.org/emergencies/ebola/75941_81198.html), our findings show that the control measures of case identification and contact tracing individually were not reaching target levels of 100% and thus need improvement to assist in termination of this outbreak in Guinea.
